# Lack of relationship between PROX1 expression and clinicopathological parameters and prognosis in gastric cancer patients: a meta-analysis and TCGA analysis

**DOI:** 10.1186/s12876-022-02229-6

**Published:** 2022-03-27

**Authors:** Zirui Jia, Yuhang Wang, Jiacheng Gao, Guo Zu

**Affiliations:** 1 Department of Gastrointestinal Surgery, The Dalian Municipal Central Hospital Affiliated of Dalian Medical University, No. 826 Southwest Road Shahekou District, Dalian, 116033 People’s Republic of China; 2 Department of Graduate School, Dalian Medical University, Dalian, People’s Republic of China

**Keywords:** PROX1, Clinicopathological parameters, Prognosis, Gastric cancer, Meta-analysis, TCGA analysis

## Abstract

**Background:**

The relationship between PROX1 expression and clinicopathological characteristics and prognosis in patients with gastric cancer (GC) remain controversial. The aim of this study is to determine the clinicopathological and prognostic significance of PROX1 expression in patients with GC.

**Methods:**

A systematic literature search and meta-analysis were performed. Odds ratio (OR) and confidence interval (CI) were used to evaluated the relationship between PROX1 expression and clinicopathological characteristics and overall survival (OS) of GC patients. Additionally, the Cancer Genome Atlas (TCGA) and the Genotype-Tissue Expression (GTEx) datasets were utilized to examine the relationship between PROX1 expression and clinicopathological significance and OS in GC patients.

**Results:**

A total of 8 studies pooling 1289 GC patients were included in the assessment. In GC patients, PROX1 expression was not related to gender (OR: 1.234, 95% CI 0.958–1.590, P = 0.104), depth of tumor invasion (OR: 0.742, 95% CI 0.428–1.287, P = 0.289), lymph node metastasis (OR: 2.161, 95% CI 0.808–5.779, P = 0.125), TNM stage (OR: 1.324, 95% CI 0.572–3.066, P = 0.513), tumor size (OR: 0.889, 95% CI 0.502–1.576, P = 0.687), distant metastasis (OR: 1.096, 95% CI 0.470–2.555, P = 0.763). In addition, we also found that PROX1 expression was not associated with 1-year OS (OR: 0.908, 95% CI 0.631–1.306, P = 0.602), 3-year OS (OR: 1.234, 95% CI 0.482–3.160, P = 0.661) and 5-year OS (OR: 0.853, 95% CI 0.266–2.736, P = 0.790). According to TCGA, in comparison with high and low PROX1 expression in GC patients, the OS did not differ statistically (p = 0.119).

**Conclusion:**

The expression of PROX1 was shown to lack a significant relationship to gender, TNM stage, depth of invasion, tumor size, stage, distant metastasis, or lymph node metastasis in statistically. The expression of PROX1 was not related to OS and it failed to be a meaningful biomarker to prevent and diagnose GC.

**Supplementary Information:**

The online version contains supplementary material available at 10.1186/s12876-022-02229-6.

## Introduction

As a type of primary cancer worldwide, gastric cancer (GC) is ranked fifth for incidence and fourth for mortality, which incidence rates are twice as high in men than in women [1]. GC imposed a significant burden on personal health as well as societies and economies. Although the application of physical examination and gastroscopy has improved the detection rate of early GC over the past decade, the majority of GC patients are at an advanced stage when they have been diagnosed [2, 3]. Advanced gastric cancer (AGC) has a poor prognosis. A distant tumor metastasis results in a poor clinical outcome for patients [4]. So a biomarker that enables earlier diagnosis and prognostic of GC is urgently needed.

Prospero-related homeobox 1 (PROX1), a vertebrate homologue of Drosophila prospero, is a homeobox gene that encodes a transcription factor and a divergent homeodomain protein [5]. PROX1 plays a pivotal role in various developmental processes of many organisms, and PROX1 signaling controls cell proliferation, differentiation and apoptosis [6]. According to a rising number of studies, PROX1 expression has been linked to carcinogenesis and prognosis in recent years. The PROX1 can promote colon cancer development by facilitating the shift from a benign to a highly dysplastic phenotype [7]. Prox1 mediates the antiproliferative impact of IFN-γ in esophageal cancer cells and Prox1 might be a viable target for new esophageal cancer treatment methods [8]. Recently, studies have suggested that PROX1 and GC are related in clinicopathological and prognostic terms. Ueta et al. reported that the high expression of PROX1 correlates positively with advanced pathological stage, lymphatic metastasis and poor prognosis but unrelated to T stage [9]. However, Alli Laitinen et al. [10] reported that the high expression of PROX1 is irrelevant to pathological stage, lymphatic metastasis, T stage, and is correlated with a good prognosis. Hafez AM et al. also reported that high expression of PROX1 is correlated with good prognosis but relevant to pathological stage, lymphatic metastasis and T stage [12]. The relationship between PROX1 expression and clinicopathological characteristics and prognosis in GC are widely disputed and remain controversial. Thus, we performed a comprehensive meta-analysis to investigate the relationship between the expression of PROX1 and clinicopathological characteristics and prognosis in GC.

## Methods

### Search strategy

A literature search was conducted by two authors (Zirui Jia and Yuhang Wang), and if a disagreement arose, it was settled by a third author (Jiacheng Gao). We searched for articles published from database inception to August 10, 2021, by searching Pubmed, Embase, Cochrane Library, Web of science, ClinicalTrials.gov and Chinese databases (WanFang, CNKI, WeiPu and CBM). No specific restrictions were applied, such as age, gender, or language. The search method consisted of two main components, which were linked together via AND: (I) Stomach Neoplasms (e.g., Stomach Neoplasm, Gastric Cancer, Stomach Cancer), (II) prospero-related homeobox 1 (e.g., PROX1, prox-1, Prospero homeobox 1)". To search, controlled vocabulary (i.e., Medical Subject Headings [MeSH] terms) and keywords associated with either of two main components were completely utilized. The search was originally developed for PubMed and then applied to the remaining 8 databases. We also performed a manual search using the reference list of major articles which were “studies assessed for eligibility” part in flowchart about 31 studies.

### Study selection

The following studies were identified for inclusion: (I) The full text of the studies is available; (II) in GC patients, the relationship between PROX1 expression, clinicopathological characteristics or prognosis was investigated.

The following studies were identified for exclusion: (I) animal experiments; (II) cell experiments; (III) repeated studies using the same data or patients; (IV) adjuvant chemoradiation before surgery was administered to the patients; and (V) the research content is unrelated to the topic.

The Newcastle–Ottawa Scale (NOS) was used to evaluate manuscript quality. The NOS ratings ranged from 0 to 9, with a score of 6 indicating excellent quality. NOS ratings greater than 6 are regarded as excellent quality scores and will be added to our meta-analysis.

### Data extraction

Two reviewers (Zirui Jia and Yuhang Wang) independently extracted results from each eligible studies: year of publication, first author, location, study period, gender, TNM stage, depth of invasion, tumor size, stage, tumor metastasis and lymph node metastasis and prognostic overall survival (OS) in 1, 3 and 5 years. If a disagreement arose, it was settled by a third author (Jiacheng Gao).

### The Cancer Genome Atlas Analysis

The Cancer Genome Atlas (TCGA) database was used to obtain tumor RNA-seq and clinicopathological parameter information for 375 GC patients, as well as 32 pairs of mRNA expression data in normal tissue samples. Other data from 359 normal tissue samples from the stomach were obtained from The Genotype-Tissue Expression (GTEx) (https://gtexportal.org/home/datasets). Like the TCGA database, complete information on normal tissue was offered by GTEx. Statistical analyses of PROX1 expression in GC and normal tissues were performed using R software. Raw read counts were normalized using DESeq2 R package. Student's t test was used to compare PROX1 expression in the TCGA cohort. We take the median of PROX1 expression as the cut off clearly. The median and above are high-expression patients, and the median below is low-expression patients. Survival analysis uses the Kaplan–Meier method and a logarithmic test. Statistical significance is defined as a P-value of less than 0.05.

### Statistical analysis

Stata 14.0 was used to conduct meta-analysis and bioinformatic analysis was performed using R (v3.6.0) and RStudio (v1.0.153). The heterogeneity of the included studies was assessed using the q test and the I^2^ index. The fixed effects model is used to calculate the 95% confidence interval (CI) of the average difference; if I^2^ ≥ 50%, the random effects model is executed. Calculate the combined Odds Ratio (OR) (95% CI) to study the relationship between PROX1 expression and clinicopathological and prognostic parameters. The funnel chart is used to determine whether or not a publication is biased. A significant difference is defined as a p-value ≤ 0.05.

## Results

### Description of studies

**Fig. 1:**
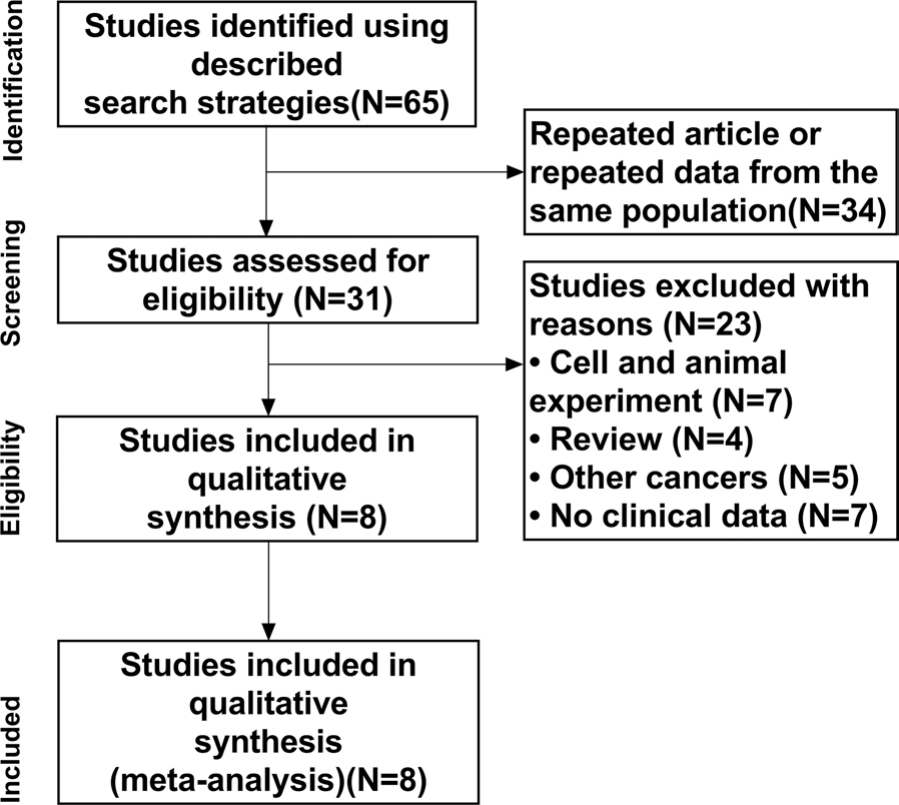
Flowchart for selection of studies

A total of 1289 patients in 8 articles were pooled in this meta-analysis (Fig. 1). We identified 65 articles from 9 database searches. 34 articles were duplicates and excluded. Among the rest of the whole 31 articles were screened for eligibility, 23 articles were excluded, including cell and animal experiments (N = 7), review only (N = 4), other cancers (N = 5) and no clinical data (N = 7). The research comprised eight studies, all in line with the survey design. Patient cases are from five countries. Table 1 summarizes the extracted data from our included studies. Studies scoring 6 or more on the NOS.

**Table 1:** Main characteristics of the eligible studies

No	First author	Year	N	Gender(M/F)	During	Country	Method (H/L)	NOS
1	Abeer M. Hafez [12]	2021	50	37/13	2014–2019	Egypt	IHC (H:5–12, L:0–4)	8
2	Zhu Li [18]	2021	110	64/46	2014–2015	China	IHC (H:4–9, L:0–3)	6
3	Congcong Min [20]	2020	85	55/30	2013–2015	China	IHC (H:4–9, L:0–3)	7
4	Aaro Kasurinen [11]	2019	275	135/140	2000–2009	Finland	IHC (H:2–3, L:0–1)	8
5	KOJI UETA [15]	2018	99	75/24	2011–2012	Japan	IHC (H:2–3, L:0–1)	7
6	Kang-Jin Park [14]	2017	327	215/112	1999–2000	Korea	IHC (H:6–9, L:0–5)	6
7	Alli Laitinen [10]	2017	273	130/143	2000–2009	Finland	IHC (H:2–3, L:0–1)	7
8	Wenan Wu [21]	2018	70	45/25	2010–2015	China	IHC (H:1, L:0)	6

*IHC* immunohistochemistry, *H* high expression, *L* low expression

### Expression of PROX1 and gender

**Fig. 2:**
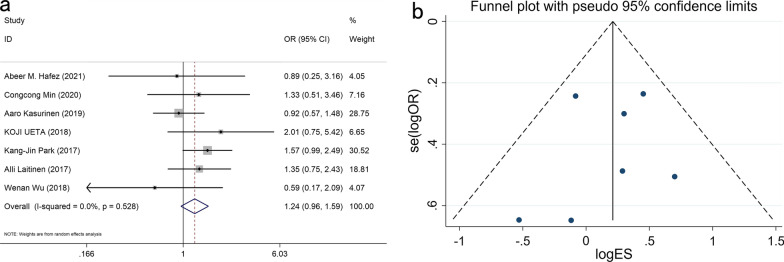
Forest plot (**a**) and funnel plot (**b**) for the relationship of PROX1 expression with gender

A total of 1179 patients from 7 studies, including 692 males and 487 females, were pooled in the analysis. Our meta-analyses found no link between PROX1 expression and gender (OR: 1.234, 95% CI 0.958–1.590, P = 0.104) (Fig. 2).

### Expression of PROX1 and tumor invasion depth

**Fig. 3:**
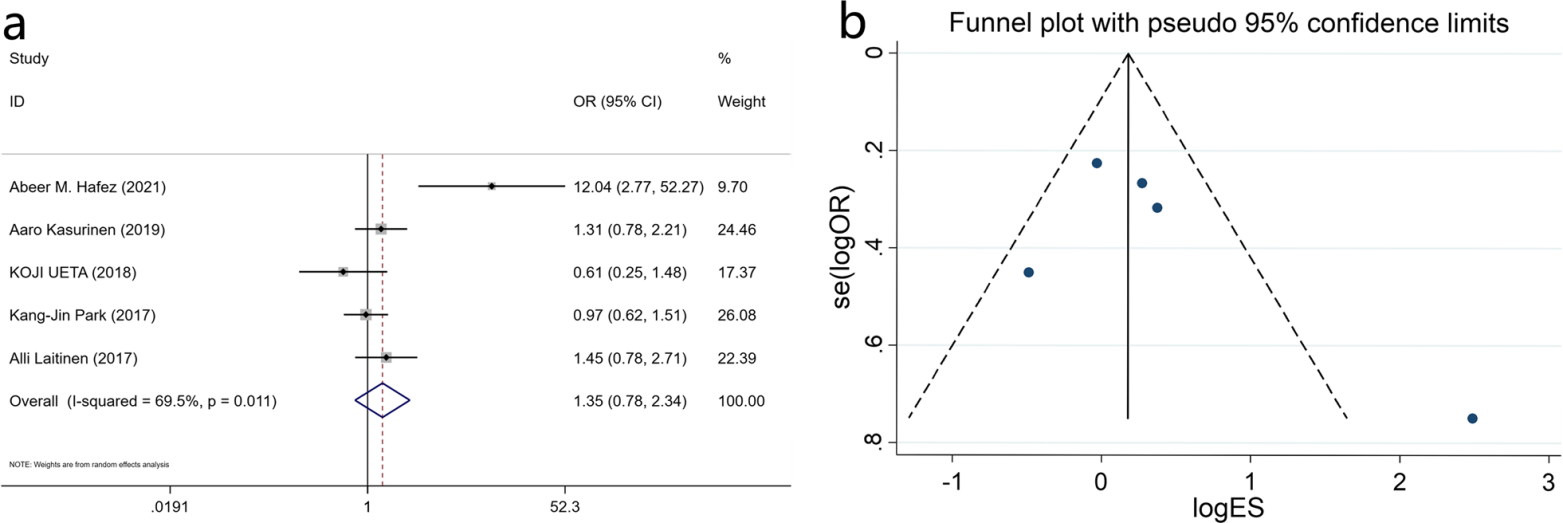
Forest plot (**a**) and funnel plot (**b**) for the relationship of PROX1 expression with the depth of tumor invasion

The meta-analysis included 1,024 patients from 5 studies to evaluate the correlation between tumor invasion depth (T1-T2 and T3-T4 groups) and PROX1 expression. PROX1 expression has no relationship with tumor invasion depth (OR: 0.742, 95% CI: 0.428–1.287, P = 0.289) (Fig. 3 and Additional file 1: Table S1).

### Expression of PROX1 and lymph node metastasis

**Fig. 4:**
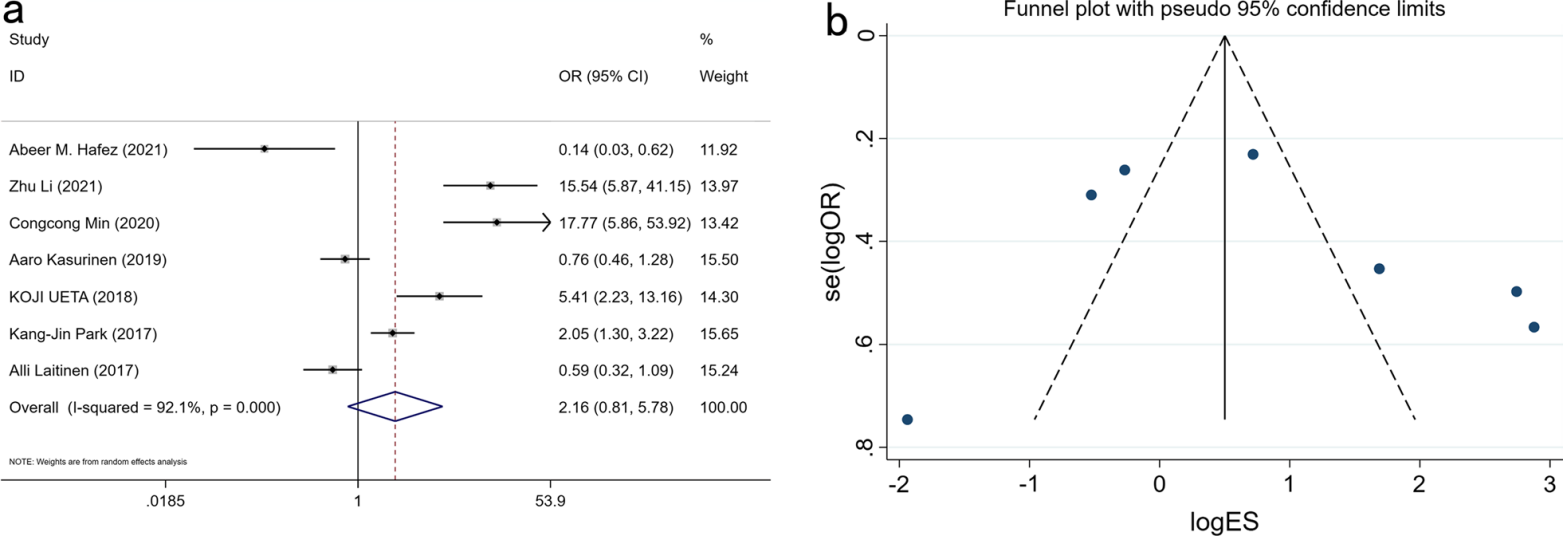
Forest plot (**a**) and funnel plot (**b**) for the relationship of PROX1 expression with lymph node metastasis

A total of 1210 GC patients from 6 studies reported the relationship between lymph node metastasis (N1-3 and N0) and the expression of PROX1 (OR: 2.161, 95% CI 0.808–5.779, P = 0.125) (Fig. 4 and Additional file 1: Table S1). Meta-analysis showed that PROX1 expression was not related to lymph node metastasis in statistic.

### Expression of PROX1 and TNM stage

**Fig. 5:**
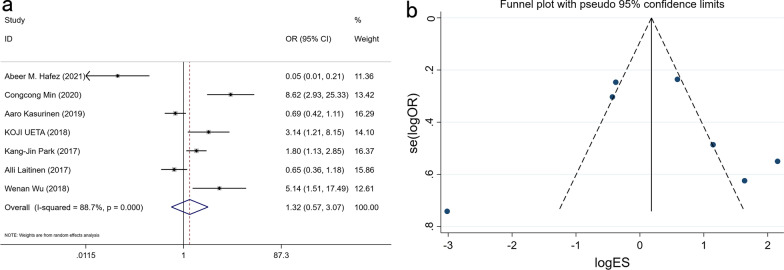
Forest plot (**a**) and funnel plot (**b**) for the relationship of PROX1 expression with TNM stage

We included 1176 patients from 7 studies in our meta-analysis and showed that the PROX1 expression was not related to the existence of TNM staging in GC (group I-II and group III-IV). (OR: 1.324, 95% CI 0.572–3.066, P = 0.513) (Fig. 5 and Additional file 1: Table S1).

### Expression of PROX1 and tumor size

**Fig. 6:**
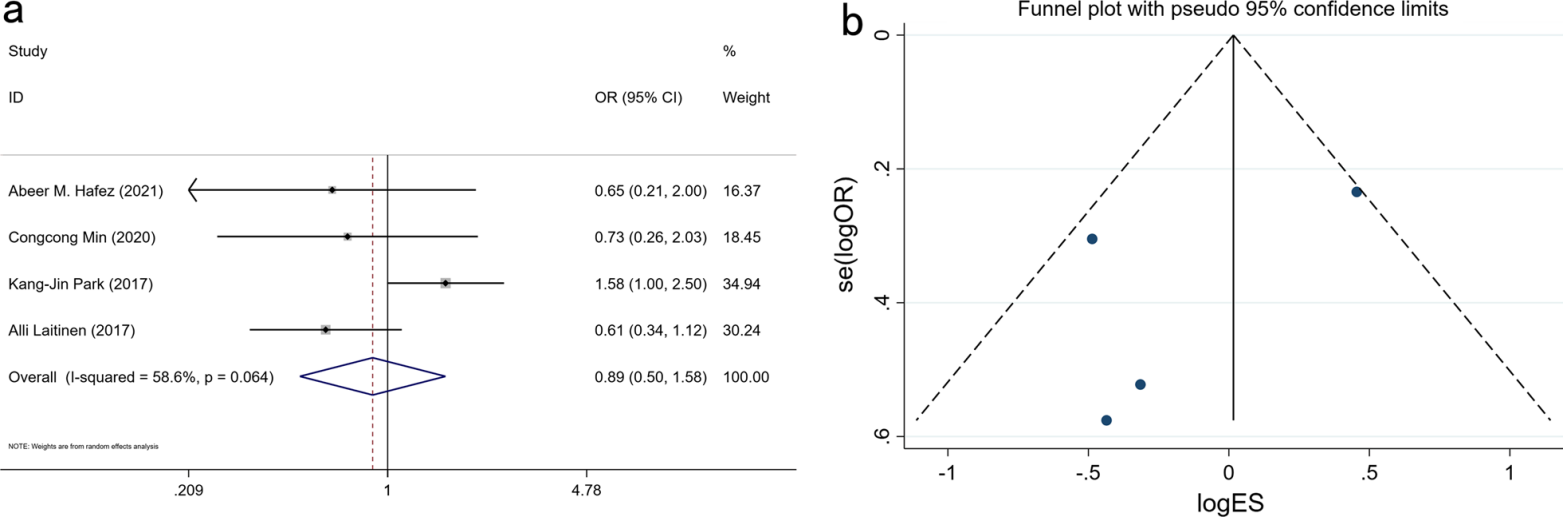
Forest plot (**a**) and funnel plot (**b**) for the relationship of PROX1 expression with tumor size

We meta-analyzed 728 patients from four studies and discovered that PROX1 expression in GC patients is not related to tumor size (≥ 5 cm and < 5 cm) (OR: 0.889, 95% CI 0.502–1.576, P = 0.687) (Fig. 6 and Additional file 1: Table S1).

### Expression of PROX1 and tumor metastasis

**Fig. 7:**
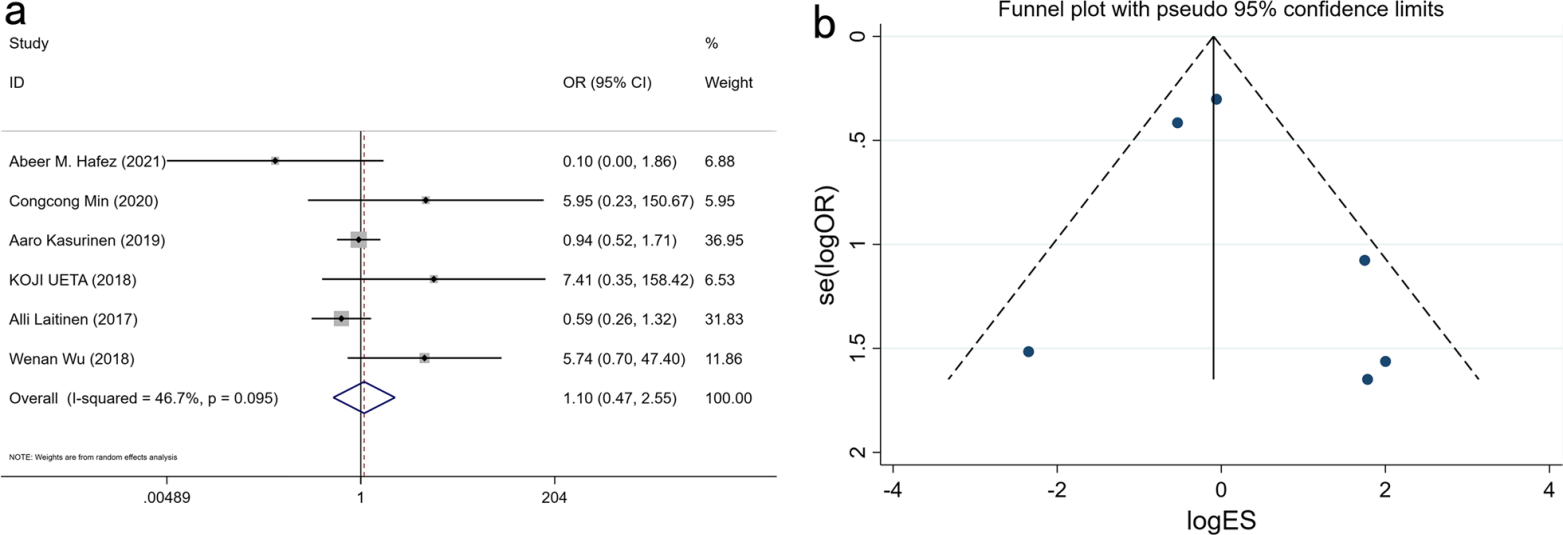
Forest plot (**a**) and funnel plot (**b**) for the relationship of PROX1 expression with metastasis

858 patients were pooled from 6 studies and the meta-analysis indicates that PROX1 expression is not related to tumor metastases (M1 and M0) in GC (OR: 1.096, 95% CI 0.470–2.555, P = 0.763) (Fig. 7 and Additional file 1: Table S1).

### Expression of PROX1 and OS

**Fig. 8:**
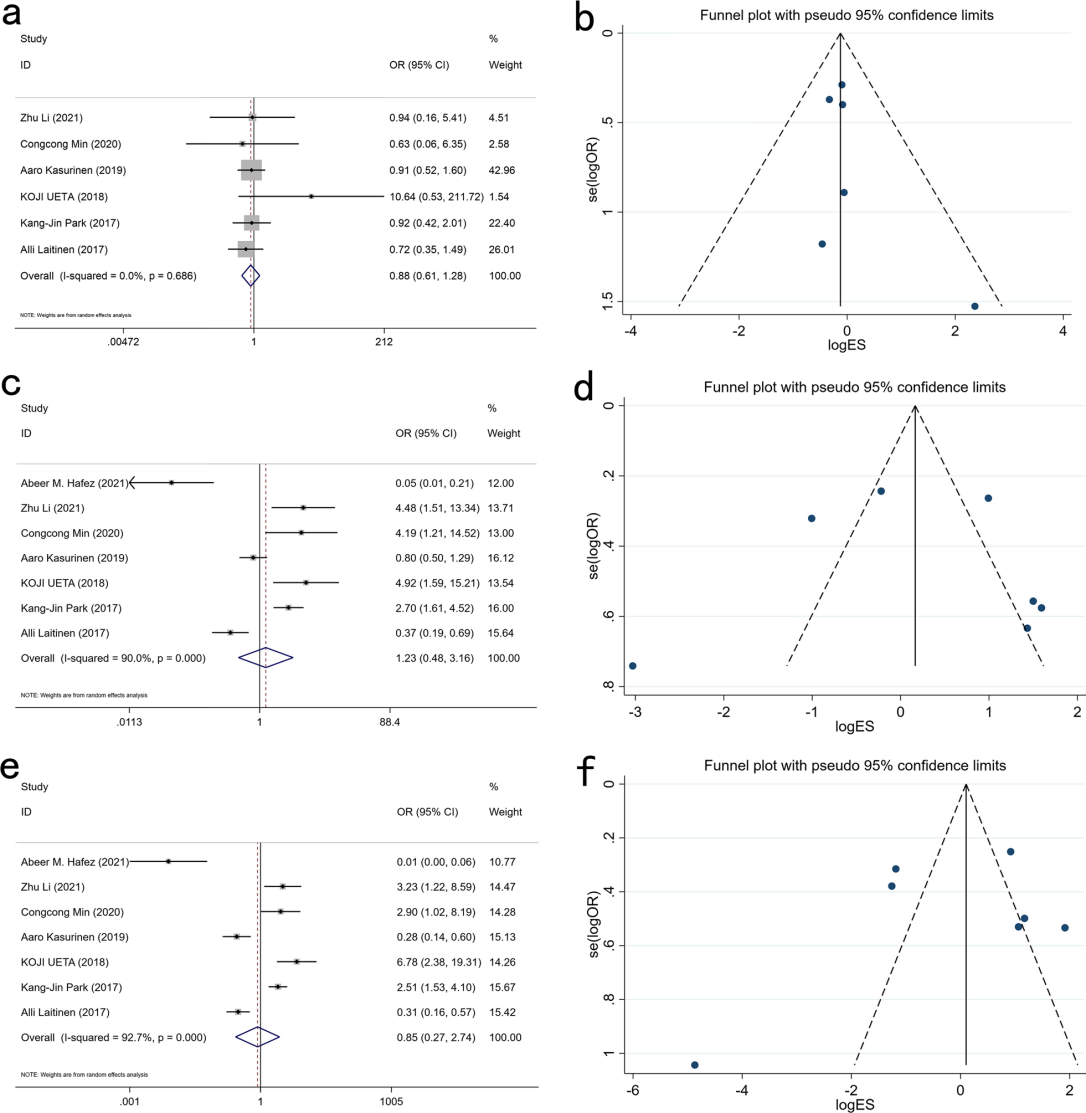
Forest plots for the association of PROX1 expression with OS in 1 (**a**), 3 (**c**) and 5 (**e**) years and funnel plots for the association of PROX1 expression with OS in 1 (**b**), 3 (**d**) and 5 (**f**) years

A total of 1231 patients from 7 studies were combined to assess the relationship between the expression of PROX1 and OS in GC patients. The findings showed that the expression of PROX1 was not related to 1-year, 3-year, and 5-year OS of GC patients (1-year OS: OR: 0.908, 95% CI 0.631–1.306, P = 0.602; 3-year OS: OR: 1.234, 95% CI 0.482–3.160, P = 0.661; 4-year OS: OR: 0.853, 95% CI 0.266–2.736, P = 0.790) (Fig. 8).

### The Cancer Genome Atlas Analysis

**Fig. 9:**
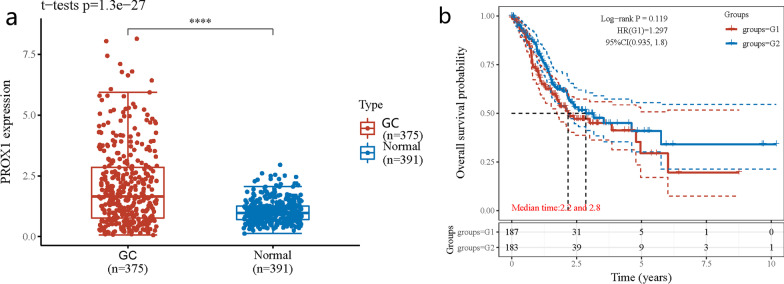
The relationship between PROX1 expression in gastric cancer (GC) patients and its prognostic value in The Cancer Genome Atlas (TCGA) cohort. **a** The amount of PROX1 expression in GC tissue versus normal gastric tissue (p < 0.001). **b** Overall survival (OS) plots of PROX1(G1: high-expression group, G2: low-expression group) in GC patients from TCGA cohort (log-rank p = 0.119). **P < 0.01 compared with the GC group

To conduct additional studies on the association between PROX1 expression and GC patients in terms of its prognostic value, we used the clinical data from TCGA and GTEx. The dataset includes 375 GC patients and 391 normal gastric control groups (Fig. 9). The contrast expressed that the expression of PROX1 was enhanced in GC patients (P < 0.001). Moreover, 370 patients with GC were divided into the PROX1 group with high expression (n = 187) and the PROX1 group with low expression (n = 183). According to TCGA, in comparison with high and low PROX1 expression in GC patients, the OS did not differ statistically (p = 0.119).

## Discussion

Previous studies have explored the relationship between PROX1 expression and clinicopathological parameters and prognosis in various cancers. Currently, a growing number of studies investigate the relationship between the expression of PROX1 and GC. However, PROX1 could be a biomarker to diagnose the GC is seriously inconsistent. Our meta-analysis showed the following: (I) the expression of PROX1 has no relationship with clinicopathological parameters of GC, (II) the expression of PROX1 has no relationship with OS.

There are several reports regarding association between the expression of PROX1 and GC. PROX1 expression could promote GC stage through a negative association with MiR-489 which was shown to suppress the formation of GC through the HDAC7 and P13k/AKT pathways [13, 22]. It is also reported that overexpression of PROX1 increased lymphatic endothelial cell invasion and tube formation by increasing VEGF-C and VEGF-D expression which may result tumor lymphangiogenesis [14]. Kang-Jin Park et al. reported a total of 327 patients finding that PROX1 expression was associated with lymph node metastases and cancer stage in a positive manner but no relation with the depth of invasion. However, the role of PROX1 in GC is controversial. Hafez AM et al. also reported that high expression of PROX1 is relevant, but oppositely, to pathological stage, lymphatic metastasis and T stage [12]. Kasurinen A et al. reported that the expression of PROX1 is not statistically correlated with the depth of invasion, lymph node metastasis and cancer stage by their study [11]. In our meta-analysis, PROX1 expression is not related to gender, TNM stage, depth of invasion, tumor size, stage, tumor metastasis or lymph node metastasis in GC.

Many studies have revealed the relationship between PROX1 expression and prognosis of GC. Kang-Jin Park et al. reported that PROX1 may be a bad promising prognostic biomarker and a novel target for GC treatment. But some other studies reported that PROX1 overexpression is associated with a better OS and acts as a good prognostic predictor for GC [10, 11]. This may be because high PROX1 expression is negatively relevant to pathological stage, lymphatic metastasis and T stage. There is ongoing controversy regarding the PROX1 expression and OS in GC patients. Our meta-analysis reveals that PROX1 expression is not related with OS at 1, 3, or 5 years. Furthermore, a comprehensive genomics-based bioinformatics analysis study that included 375 cases with OS data for GC patients also supports our viewpoint and confirms that expression of PROX1 is not associated with OS at 1-year, 3-years, and 5-year. However, in the TCGA database, we discovered that PROX1 is highly expressed in GC. This suggests that PROX1 may be operative in GC. Park KJ et al. reported that high expression of PROX1 in tumors may be associated with tumor proliferation [14]. We did not explore the relationship between PROX1 expression and GC proliferation. This requires further research.

This meta-analysis also has its limitations. First, the included studies were published only in English and Chinese. Second, the number of included studies and the total number of patients are small. Additional laboratory studies and analysis on larger, well-defined patient cohorts are required to detect the relationship between PROX1 and GC. Furthermore, the extraction cutoffs (positive/negative or high/low) of the expression of PROX1 in GC tissues are not totally same in 8 studies and this may induce heterogeneity. In addition, some data are derived from estimates of survival curves, not individual patient data. These may induce heterogeneity or bias in our results. It's possible that more studies and large sample sizes would come out in the future.

## Conclusion

We performed a meta-analysis to figure out the relationship between PROX1 expression and clinicopathological and prognostic significance in GC patients. We found PROX1 expression is not correlated with gender, TNM stage, depth of invasion, tumor size, stage, distant metastasis and lymph node metastasis. The expression of PROX1 is not associated with OS and it fails to be a meaningful biomarker to prevent and diagnose the GC. Larger sample sizes will be used in our continued research program in the future.

## Supplementary Information

**Additional file 1: Supplementary Table 1**. Results for the meta-analysis between PROX1 expression and clinicopathological parameters of GC.

## Data Availability

The meta-analysis data generated or analyzed during this study is included in this published article and its supplementary information files. The datasets used and/or analyzed during the current study are available from TCGA repository: https://portal.gdc.cancer.gov/; GTEx repository: https://www.gtexportal.org/home/.
