# Supplementary material for: Lack of relationship between PROX1 expression and clinicopathological parameters and prognosis in gastric cancer patients: a meta-analysis and TCGA analysis
Source: BMC Gastroenterol. 2022 Mar 27;22:142. doi: 10.1186/s12876-022-02229-6 (PMC8958786; doi:10.1186/s12876-022-02229-6)
Supplement: Supplementary file 1 — Additional file 1: Supplementary Table 1. Results for the meta-analysis between PROX1 expression and clinicopathological parameters of GC. [file 12876_2022_2229_MOESM1_ESM.docx]

Supplementary Table 1: Results for the meta-analysis between PROX1 expression and clinicopathological parameters of GC

| Clinical parameters | No. of studies | Overall OR (95%CI) | Heterogeneity test (Q, I^2^ ,P) |
| --- | --- | --- | --- |
| Size (≥5cm vs <5cm) | (10)(12)(14)(20) | 0.889(0.502-1.576) | 7.25, 58.6%, 0.064(random-effect) |
| Invasion depth (T1-T2 vs T3-T4 groups) | (10)(11)(12)(14)(15) | 0.742(0.428-1.287) | 13.1, 69.5%, 0.011(random-effect) |
| lymph node metastasis (N0 vs N1-3) | (10)(11)(12)(14)(15)(18)(20) | 2.161(0.808-5.779) | 76.07, 92.1%, 0.000(random-effect) |
| Tumor metastases (M1 vs M0) | (10)(11)(12)(15)(20)(21) | 1.096(0.470-2.555) | 9.38, 46.7%, 0.095(random-effect) |
| TNM stage (I-II vs III-IV) | (10)(11)(12)(14)(15)(18)(20)(21) | 1.324(0.572-3.066) | 52.97, 88.7%, 0.000(random-effect) |

Abbreviation: OR, odds rates.
